# *F1000Research*: *Tics* welcomes you to 21st century biomedical publishing

**DOI:** 10.12688/f1000research.5664.1

**Published:** 2014-11-12

**Authors:** Kevin J. Black

**Affiliations:** 1 Departments of Psychiatry, Neurology, Radiology, and Anatomy & Neurobiology, Washington University School of Medicine, St. Louis, MO, USA

## Abstract

Tics are repeated, usually suppressible movements or vocalizations. They are the defining features of tic disorders including Tourette syndrome, but many people have them for shorter durations at some point in childhood. This editorial marks the beginning of the *F1000Research: Tics *specialty section, an effort to provide a single portal to modern research on tics and tic disorders. Publications in *F1000Research: Tics* benefit from *F1000Research*’s novel approach to publishing, in which articles can be published within days of submission. Peer review happens after publication and is fully open. When the submitted article or a revision is approved, it is promptly submitted to repositories including NIH’s PubMed Central. In addition to research articles and reviews, *F1000Research: Tics* will publish study protocols, clinical practice articles, case reports, and data notes. The home page will also provide links to expert recommendations of articles that have appeared elsewhere, and to relevant posters from scientific meetings (http://f1000.com/posters/). *F1000Research*’s approach is enabled by the capabilities of internet publication, including space to publish the full results of a study rather than just a few graphs selected from the data. Publishing methodologically sound studies without requiring subjective editorial judgments of novelty or broad appeal brings numerous advantages, including minimizing publication bias and shining the light of openness on peer review. To celebrate the launch of the Tics section, *F1000Research* is offering discounted article processing charges for manuscripts submitted by March 1st 2015. I have had good experiences publishing in *F1000Research*, and look forward to seeing a wide range of tic-related manuscripts submitted.

## Editorial

### Tics

Tics are the defining feature of Tourette syndrome, and may affect as many as half of all people at some point during childhood. Tics range from unobtrusive movements and noises that go unnoticed or are misattributed to allergies or restlessness, to complex patterns of movement or words that may be so frequent, severe or uncomfortable as to be disabling ^1^. Tics have fascinated physicians, psychologists, historians and neuroscientists, in part because so many tics initially appear to be normal movements when seen in isolation, and in part because most tics are temporarily suppressible with effort and concentration.

### Why *F1000Research: Tics*?

Many journals have published research on tic disorders, but hitherto none has focused exclusively on tics. This editorial marks the beginning of the *F1000Research: Tics* specialty section, an effort to provide a single portal to modern tic research. *F1000Research: Tics* will publish research articles and reviews, but also study protocols, clinical practice articles, case reports, and data notes. All these flow through the *F1000Research* submission system and benefit from *F1000Research*’s novel approach to publishing, in which articles can be published within days of submission. Fully open peer review happens after publication. The article collection will also highlight both expert article recommendations of articles that have appeared elsewhere, and relevant posters from scientific meetings ( http://f1000.com/posters/).

### Welcome to 21st century publishing

*Le roi est mort, vive le roi!*

In the not-so-distant days of my residency training, staying abreast of relevant literature included paying for paper journals and skimming through them on the occasional call night when there was time to breathe. The word “paper” is important in that sentence, because the format required numerous compromises. Only so many articles could be published. Each article could take only so many pages. It was a given that a publication could contain only a bare fraction of the results. As a consequence, some journals had to reject most papers submitted to them, and could select the papers that would bring a wider audience or greater notoriety.

Well, paper is dead. Or if it is not dead, it is lingering like a heroine in a tragic opera whose breath support in the face of a mortal wound mystifies all but the most enthusiastic suspenders of disbelief. Nowadays when I want to find a paper on a given topic, I open a web browser rather than a paper journal. Yet compromises born of the limitations of a paper journal linger on like the vermiform appendix: some publishers remain set on publishing a set number of pages of terse reports with each issue.

Scientific publishing of course is not dead. A myriad of online journals seem to have sprung up over the past decade or so. Although some appear to be destined for at most ephemeral interest, internet publishing has led to several important advances. The most important of these may be free access to relevant literature for patients and for scientists or clinicians from third-world countries. The most obvious advantage of internet publishing is the opening up of space: as the most prominent example, *PLOS ONE* published its 100,000th article before reaching its eighth birthday—without imposing limits on words, figures or pages. Somewhat counterintuitively to many raised on paper journals, the avalanche of publications in the past 10 years has improved the overall quality of the scientific literature, not only because of the many outstanding reports included in the total but also by mitigating publication bias.

**Box 1. Tic research already on F1000.com**

***F1000Research***

- **RESEARCH ARTICLE:** A pilot study of basal ganglia and thalamus structure by high dimensional mapping in children with Tourette syndrome ( http://f1000research.com/articles/2-207/v1)
- **DATA NOTE:** A revised method for measuring distraction by tactile stimulation ( http://f1000research.com/articles/3-188/v1)

***F1000Posters***

- **POSTER:** A voxel-based morphometry study of Tourette syndrome (Annual meeting, American Neuropsychiatric Association, Feb 2003) ( http://f1000.com/posters/browse/summary/1092954)
- **POSTER:** A preliminary test of the mnestic hypometria hypothesis in Parkinson disease (Annual meeting, Movement Disorders Society, Jun 2010) ( http://f1000.com/posters/browse/summary/1092989)
- **POSTER:** A preliminary study of quantitative temporal modeling of the urge to blink during blink suppression (Washington University School of Medicine 8th Annual Research Training Symposium and Poster Session, Aug 2013) ( http://f1000.com/posters/browse/summary/1095804)
- **POSTER:** http://f1000.com/posters/search?query=Tourette

***F1000Prime***

- http://f1000.com/prime/1164614
- http://f1000.com/prime/search/recommendations?query=Tourette&sortBy=f1000Factor

Reviewers for *F1000Research* are instructed to focus solely on whether the methods are appropriate and the results justify the conclusions. Novelty or a subjective prediction of impact are not required. Properly designed and performed studies with negative results are important to the scientific literature; their publication helps minimize the “file-drawer effect,” *i.e.* the skewed view of a scientific question that results when negative studies are less likely to be published than positive studies ^2– 4^. Of course a specialty area like tic disorders benefits when predicted interest to a broad audience is not required.

The nearly unlimited space on the internet also allows abolishing paper relics like color figure charges, arbitrary limits on the number of figures, and the relegating of information needed for review to a separate supplement. This brings up a benefit that I have only recently learned to appreciate: open data.

### Open data

*F1000Research* requires publication of the *data*, not just a graph or two summarizing the data. This requirement is new to some, but increasingly funders like NIH and the Wellcome Trust also mandate releasing data from research they support ^5^. This move is supported by many scientists ^6, 7^ and some publishers ^8– 10^. One obvious reason for data publication is that many kinds of research generate more data than a table or plot can adequately summarize. As Tom Nichols recently wrote:

“[A]n fMRI study requires hundreds of man-hours, costly scanner time, and laborious data analysis to process gigabytes of image data. Yet, what is the quantitative result that is the core of a published paper? A list of *x*, *y*, *z* brain atlas coordinates of activation, a dataset that can be recorded on a Post-it note! While figures may show the pattern of brain activation, if any quantitative result (point estimate ± standard error) is given, it is only for a selected region of the brain. Is it really acceptable practice that, of the gigabytes of raw and processed data that are generated, only [kilobytes] of data are ultimately shared?” ^11^

Other researchers may think of ways of looking at the data that the submitter had never considered. For instance, last year *F1000Research* debuted a data visualization tool by which the reader could replot tabular data at will without leaving the online article (see the Data Plotter in ^12^). Open data brings unexpected benefits as well. For instance, publications with full data release are more highly cited ^13^. This may relate to the observation that authors willing to share data had higher quality data and more appropriate analyses ^14^. Additionally, data sharing preserves information to enable future meta-analyses and other uses. For example, recently I have been reviewing the literature on the first few months of the course of tic disorders. Many times I encountered studies that had collected data I was interested in, but the report was focused on other results and the information I wanted was omitted. In one case, even a full-length monograph had to make choices about which data would appear in tables and text, and omitted the data I was seeking. Finally, anyone who has been doing science long enough may have lost large data sets, or found them on a medium that had become corrupted or was no longer convenient to access. In these scenarios, publication of the full data would have benefited the original researcher as much as anyone else, especially since permanently self-archiving data carries ongoing costs that may not be supported by grants that end after a few years.

### *F1000Research*’s specific advantages

Some of these advantages are shared by numerous publications. But *F1000Research* brings additional advantages related to its strategy of post-publication open review. I for one am heartily weary of the delay and frustrations that come from having a submitted manuscript rejected for reasons of interest and breadth alone, addressing sometimes uninformed or even snarky unsigned reviews, adapting to a new journal’s word limits, reformatting, and waiting for the cycle to begin again. Or consider that when an experiment ends, traditionally it has been unreasonable to expect to publish it in time to cite it in a grant application due next month. By contrast, with *F1000Research: Tics* it is quite possible.

More importantly, open data and open reviews address a real and topical problem in science: reproducibility ^15^. This is a problem across a wide range of journals, and in fact highly selective journals tend to have higher, not lower, retraction rates ^16– 18^. In part this is likely due to the fact that “man bites dog” (or a paradigm-busting new result) makes great news copy but is less likely to be replicated consistently ^19^. Open reviews, along with open data, allow the scientific community to assess the quality of the reviews as well as the quality of the submission. One recent example came from a report claiming that remote effects of one person’s experience could be detected on another person’s EEG: silent rejection of the manuscript, or publication without open data and review, would not have been nearly so instructive to the scientific community as the thoughtful comments and re-analysis of the primary data by a peer reviewer ^20, 21^.

### Measuring impact

Most readers will be familiar with the journal impact factor, a journal-level citation metric that has some validity ^17, 22^. However, in addition to other failings— *e.g*., it does not correlate significantly with statistical power ^17, 23^—the impact factor was never intended to measure the impact (much less quality) of individual papers. Numerous prominent scientists, scientific societies and publishers have signed The San Francisco Declaration on Research Assessment (DORA), which calls for an end to using journal-level metrics to judge the quality of an individual’s scientific contributions ^24^. Since online searches for individual papers became widely accessible, the citation rate for a given paper has become less tightly associated with the impact factor of the journal in which it appeared ^25^.

Publishing all appropriate contributions regardless of predicted breadth or novelty, and publishing Data Notes, study protocols, case reports, and commentary in addition to traditional research articles and reviews, are strategies aimed at improving the scientific literature rather than at inflating a journal-level metric. Some of the publications will be “hot” new science seeking rapid publication so as not to be scooped. But if some other publications are of lower quality or novelty, at least they are accompanied by the full data set and open reviews, so readers can judge the merits independently (or even contribute to the discussion).

### Novelty

“You must try new things. … If you don’t try, you won’t learn. If you don’t learn, you won’t succeed”. —Mark Willes ^26^

Anything new will always bring some fear. On initial exposure to *F1000Research*’s publication model, it may be natural to hesitate at making one’s work publicly available, “warts and all,” before critique from one’s peers. Similarly, opening the reviews of one’s work for all to read may sound scary. Yet physicists and mathematicians have gotten along fine posting pre-reviewed manuscripts on arXiv ^27, 28^, and the simple requirement that peer reviews be signed seems to produce a remarkable tempering effect on the tone of critical comments. I have had good experiences publishing in *F1000Research*, and look forward to more with *F1000Research: Tics*.

### How it works

Submitting to *F1000Research: Tics* will be straightforward. Detailed author guidelines are available here ( http://F1000Research.com/author-guidelines#oep). During submission, the author simply indicates that the submission is intended for the *Tics* section. After initial quality control, submissions are published immediately and sent out for peer review. Peer review reports are signed and receive individually citable DOIs. For instance, a Data Note from my lab led to a thoughtful review that added important context and can be cited independently ^29^. Articles not approved by initial peer review can be revised without additional fees and re-reviewed. Once approved, the current version is promptly submitted to PubMed Central without embargo, a real benefit to authors funded by NIH and several other funding bodies. Importantly, I do not select reviewers or control publication for *F1000Research: Tics*. I do get to choose whether to accept a submission into the *Tics* collection, but excluded articles will remain eligible for *F1000Research*. I will aim initially to exclude only for topical irrelevance, in keeping with *F1000Research*’s philosophy.

### Join the fun! (Call for papers)

I will be submitting several papers to *F1000Research: Tics*, and invite manuscripts from other authors addressing all aspects of tics and tic disorders. All of *F1000Research*’s article types are welcome, from case reports and opinion articles through software tools and study protocols to exciting results from large research studies ( http://F1000Research.com/author-guidelines#f1000at). I encourage organizers of professional meetings to invite speakers and poster authors to submit posters or slides to *F1000Posters* (at no charge) or full articles to *F1000Research: Tics*. The article processing fees are modest, and *F1000Research* is celebrating the launch of the *Tics* section with an initial discount on articles submitted successfully to the collection by March 1st 2015. Welcome to *F1000Research: Tics*!
